# Emergency angiography for trauma patients and potential association with acute kidney injury

**DOI:** 10.1186/s13017-021-00400-0

**Published:** 2021-11-04

**Authors:** Ryo Yamamoto, Ramon F. Cestero, Jo Yoshizawa, Katsuya Maeshima, Junichi Sasaki

**Affiliations:** 1 Trauma Service/Department of Emergency and Critical Care Medicine, Keio University School of Medicine, 35 Shinanomachi, Shinjuku, Tokyo 160-8582 Japan; 2 Department of Surgery, UT Health San Antonio, San Antonio, TX USA

**Keywords:** Angiography, Embolization, Hemostasis, Acute kidney injury

## Abstract

**Background:**

Angiography has been conducted as a hemostatic procedure for trauma patients. While several complications, such as tissue necrosis after embolization, have been reported, little is known regarding subsequent acute kidney injury (AKI) due to contrast media. To elucidate whether emergency angiography would introduce kidney dysfunction in trauma victims, we compared the incidence of AKI between patients who underwent emergency angiography and those who did not.

**Methods:**

A retrospective cohort study was conducted using a nationwide trauma database (2004–2019), and adult trauma patients were included. The indication of emergency angiography was determined by both trauma surgeons and radiologists, and AKI was diagnosed by treating physicians based on a rise in serum creatinine and/or fall in urine output according to any published standard criteria. Incidence of AKI was compared between patients who underwent emergency angiography and those who did not. Propensity score matching was conducted to adjust baseline characteristics including age, comorbidities, mechanism of injury, vital signs on admission, Injury Severity Scale (ISS), degree of traumatic kidney injury, surgical procedures, and surgery on the kidney, such as nephrectomy and nephrorrhaphy.

**Results:**

Among 230,776 patients eligible for the study, 14,180 underwent emergency angiography. The abdomen/pelvis was major site for angiography (10,624 [83.5%]). Embolization was performed in 5,541 (43.5%). Propensity score matching selected 12,724 pairs of severely injured patients (median age, 59; median ISS, 25). While the incidence of AKI was rare, it was higher among patients who underwent emergency angiography than in those who did not (140 [1.1%] vs. 67 [0.5%]; odds ratio = 2.10 [1.57–2.82]; *p* < 0.01). The association between emergency angiography and subsequent AKI was observed regardless of vasopressor usage or injury severity in subgroup analyses.

**Conclusions:**

Emergency angiography in trauma patients was probably associated with increased incidence of AKI. The results should be validated in future studies.

**Supplementary Information:**

The online version contains supplementary material available at 10.1186/s13017-021-00400-0.

## Background

Angiography has been conducted as a hemostatic or diagnostic procedure for trauma patients for several decades, and indications for angiography after injury continue to expand [1, 2]. While surgery is the standard treatment for patients with bleeding, angiography with embolization to control arterial hemorrhage has been performed as a less invasive procedure [1, 3, 4]. Notably, the success rate of non-operative management for patients with high-grade splenic injury has been reported to be improved to 95% after emergency angiography with embolization [5, 6].

While several complications of angiography including tissue necrosis, re-bleeding, and vascular injury [7, 8] have been reported, little is known regarding subsequent kidney injury due to contrast media that is used in angiography for trauma victims. Acute kidney injury (AKI) following intravascular administration of contrast media is defined by several terms such as contrast-induced nephropathy (CIN), contrast-induced AKI (CI-AKI), or post-contrast AKI (PC-AKI) [9, 10], and nearly 10% of patients who were given considerable amount of contrast media in percutaneous coronary angiography were found to develop PC-AKI [11]. Conversely, a recent systematic review identified that a relatively small amount of contrast media, such as the amount administered in a contrast-enhanced CT scan, was not associated with newly developed AKI unless baseline kidney function was severely compromised [12]. Since the dosage of contrast media administered during emergency angiography for trauma patients is often higher than the amount given for a CT with contrast [13], risks of angiography on kidney function exist, and this potential effect has not been extensively examined among severely injured patients. It should be also emphasized that some trauma patients who undergo angiography are likely at higher risk for AKI due to hemodynamic instability and direct traumatic insult to the kidney [14, 15].

Accordingly, to eventually ascertain whether emergency angiography might independently introduce subsequent AKI among trauma patients, we used a nationwide trauma database to compare the incidence of AKI between patients who underwent emergency angiography and those who did not. We hypothesized that emergency angiography would be independently associated with higher incidence of AKI in severely injured trauma patients.

## Methods

### Study design and setting

We conducted a retrospective cohort study using data from the Japan Trauma Data Bank (JTDB). The JTDB was established as a Japanese nationwide trauma registry in 2003, representing > 250 participating hospitals and tertiary care centers. Before initiating the study, all collaborating hospitals obtained individual local institutional review board approval for conducting research with human subjects [16].

Current practice in Japan recommends consideration for emergency angiography in hemodynamically stable trauma patients who have evidence of bleeding or severe organ injury diagnosed with contrast-enhanced CT scan. The decision to perform emergency angiography is determined by discussion between trauma surgeons and radiologists based on CT scan findings and patient status. However, as trauma surgeons are not always present in the hospital, emergency physicians sometimes independently decide emergency angiography.

### Study population

We retrospectively reviewed data from the JTDB between January 2004 and March 2019. Trauma patients who were aged ≥ 18 years and were transported directly from the scene were included. Patients who arrived with cardiac arrest and those with missing data on the emergency angiography were excluded.

### Data collection and definitions

Available data included age, sex, mechanism of injury, comorbidities, vital signs on hospital arrival, Abbreviated Injury Scale (AIS) score, Injury Severity Score (ISS), presence of compartment syndrome in extremities, vasopressor usage, and any surgical procedures including laparotomy, thoracotomy, resuscitative thoracotomy (RT), and resuscitative endovascular balloon occlusion of the aorta (REBOA). Procedures performed related to traumatic kidney injury such as total nephrectomy, partial nephrectomy, and nephrorrhaphy were also available. The target regions for angiography and concomitant embolization were recorded in the database, but details regarding the indications for angiography or the hemodynamic status during angiography were not available.

Emergency angiography was recorded when an angiography was urgently performed as a hemostatic or diagnostic procedure during the initial resuscitation, regardless of preceding hemostatic surgery or time interval between hospital arrival and initiation of the angiography. Any scheduled or unscheduled angiographic procedures which were conducted after the achievement of initial resuscitation with hemostasis (e.g., angiography for re-bleeding or pseudoaneurysm found later), were not recorded as emergency angiography.

AKI was diagnosed by treating physicians based on a rise in serum creatinine and/or fall in urine output according to any published standard criteria depending on the time of study inclusion. Predefined uniform criteria for AKI were not used in the database. Serum creatine values, urine output, and hemodialysis requirement were not available in the database.

### Outcome measures

The primary outcome was the incidence of AKI prior to discharge. Secondary outcomes included hospital-free days and intensive care unit (ICU)-free days until day 30.

### Statistical analysis

Patient data were divided between angiography and non-angiography groups. The angiography group consisted of patients who underwent emergency angiography, while the non-angiography group consisted of those who were treated without emergency angiography. Unadjusted analysis was performed on the primary outcome with Chi-square test.

To select a similar cohort of control patients from the non-angiography group, propensity score matching was performed [17]. The propensity score was developed using a logistic regression model to estimate the probability of being assigned to the angiography group [18]. Relevant covariates were selected from known or possible indications for angiography and background risks for kidney injury, including baseline characteristics such as age, sex, comorbidities, mechanism of injury, vital signs on admission, abdominal AIS, renal AIS, ISS, surgical procedures, and the type of surgery on the kidney [9, 10, 13–15, 19]. Patients with missing covariates were excluded from propensity score calculation. The precision of discrimination of propensity score was analyzed with the c-statistic. One-to-one propensity score matching was then performed using a greedy matching algorithm without replacement, where a caliper width of less than 0.2 of the standard deviation of logit-transformed propensity score was applied. Equality of patient characteristics between both groups after matching was evaluated with the standardized difference of each covariate, in which standardized difference < 0.1 was considered as non-biased distribution [18, 20]. The inter-group comparison of primary and secondary outcomes after propensity score matching was performed using Chi-square tests or ordinal regression analysis, as appropriate.

An inverse probability weighting analysis using propensity score and a logistic regression analysis with propensity score as covariate were conducted as sensitivity analyses on the whole population [17, 21]. Furthermore, the primary outcome was compared between the angiography and non-angiography groups in the subgroup of patients who were divided based on the presence of chronic kidney disease (CKD) before injury, age (≥ 65 vs < 65 years), severity of injury (ISS ≥ 25 vs < 25), vasopressor usage, and the year of injury (2004–2009 vs 2010–2019).

Descriptive statistics are presented as the median (interquartile range) or number (percentage). Results are shown using standardized difference and 95% confidence interval (CI). Missing/ambiguous values were used without manipulation. Testing of the hypothesis was only performed on the primary outcome, in which a 2-sided α threshold of 0.05 was considered statistically significant. All statistical analyses were conducted using SPSS, version 26.0 (IBM, Armonk, NY), and Microsoft Excel (Microsoft, Redmond, WA).

## Results

### Patient characteristics

**Fig. 1:**
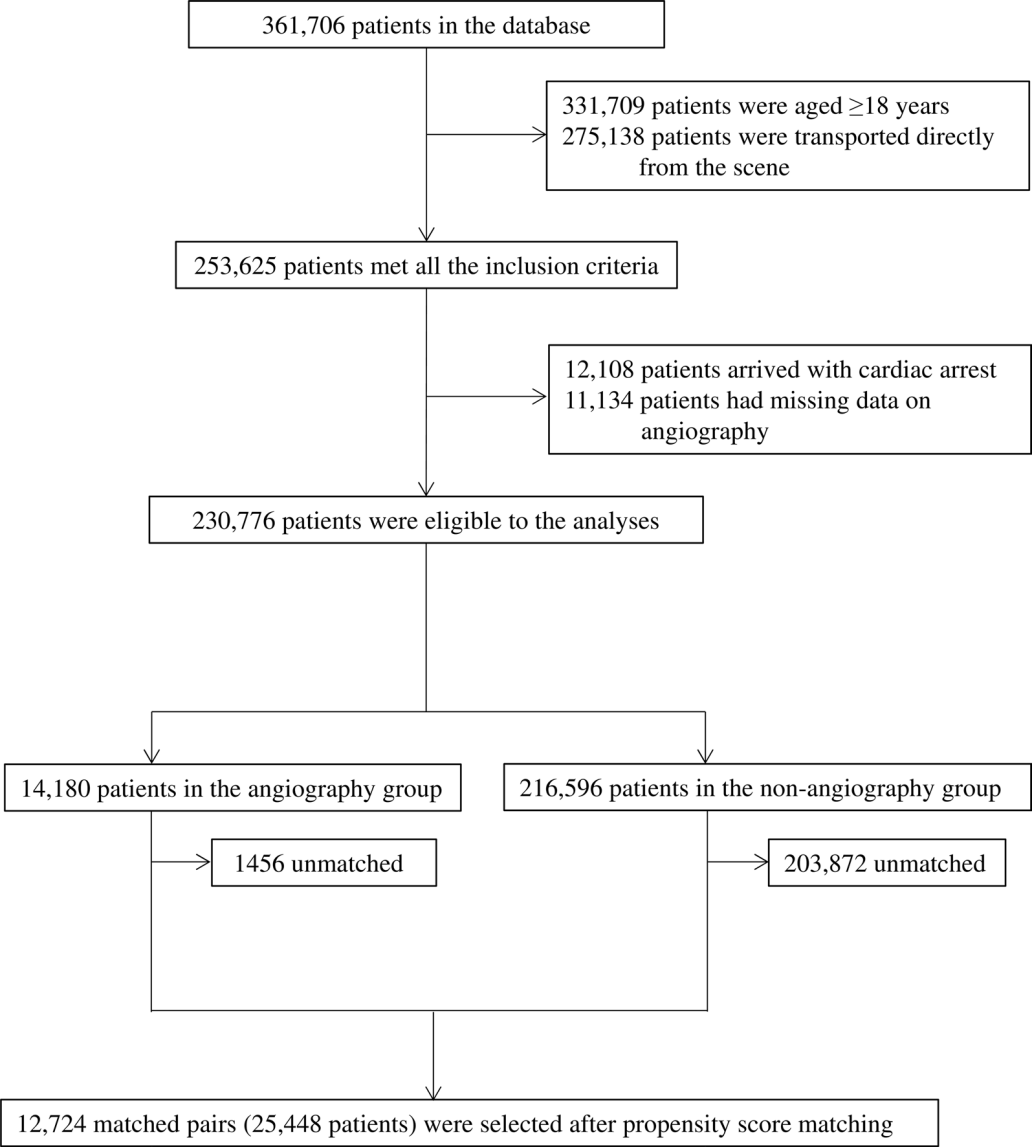
Patient flow diagram. Among 361,706 trauma patients in the database, 331,709 adult patients were transported directly from the scene, and 230,776 patients were eligible for this study. A total of 14,180 (6.1%) patients underwent an emergency angiography. Among the 14,180 patients in the angiography group, 12,724 patients were matched with controls in the non-angiography group

Among 361,706 trauma patients in the database, 331,709 adult patients were transported directly from the scene, and 230,776 patients were eligible for this study (Fig. 1). A total of 14,180 (6.1%) patients underwent an emergency angiography.

Patient characteristics are summarized in Table 1. Patients in the angiography group were younger and had lower Glasgow Coma Scale (GCS) and lower systolic blood pressures (sBP) on arrival compared with those in the non-angiography group, as well as higher ISS (25 [16–35] vs. 10 [9–18]). Furthermore, more patients in the angiography group underwent surgical procedures than those in the non-angiography group, including total nephrectomy (43 [0.3%] vs. 100 [0.0%]). Vasopressors were used more in the angiography group than in the non-angiography group. Target regions for emergency angiography are shown in Table 2. The abdomen/pelvis was the major site for angiography (10,624 [83.5%]), and embolization was performed in 5,541 (43.5%) patients.

**Table 1:** Characteristics of patients with or without angiography

	Before matching				After matching				
	Angiography	No angiography	*p* value	Standardized difference	Angiography		No angiography		Standardized difference
*Case*	14,180	216,596			12,724		12,724		
Age, years, median (IQR)	59 (38–74)	63 (43–78)	< 0.001	0.068	59 (38–74)		58 (53–60)		0.090
Mechanism of Injury, blunt, n (%)	13614 (97.4%)	199683 (96.1%)	< 0.001	0.075	12392 (97.4%)		12196 (95.9%)		0.085
Missing data	202 (1.4%)	8748 (4.0%)							
*Comorbidity, n (%)*									
Coronary artery diseases	366 (2.6%)	7882 (3.6%)	< 0.001	0.061	339 (2.7%)		215 (1.7%)		0.067
Heart failure	145 (1.0%)	4104 (1.9%)	< 0.001	0.073	132 (1.0%)		65 (0.5%)		0.060
Cerebrovascular diseases	465 (3.3%)	10844 (5.0%)	< 0.001	0.087	415 (3.3%)		287 (2.3%)		0.061
Diabetes	1126 (7.9%)	22660 (10.5%)	< 0.001	0.087	1019 (8.0%)		985 (7.7%)		0.010
Hypertension	2511 (17.7%)	51309 (23.7%)	< 0.001	0.148	2306 (18.1%)		1896 (14.9%)		0.087
CKD	95 (0.7%)	2906 (1.3%)	< 0.001	0.067	83 (0.7%)		55 (0.4%)		0.030
*Vital signs on hospital arrival*									
BP - systolic, mmHg, median (IQR)	118 (91–141)	139 (120–160)	< 0.001	0.451	118 (92–142)		119 (94–143)		0.003
missing data	162 (1.1%)	3506 (1.6%)							
GCS, median (IQR)	14 (11–15)	15 (14–15)	< 0.001	0.289	14 (12–15)		14 (11–15)		0.026
Missing data	554 (3.9%)	13903 (6.4%)							
*Injury severity*									
ISS, median (IQR)	25 (16–35)	10 (9–18)	< 0.001	0.913	25 (16–34)		24 (16–34)		0.056
Missing data	290 (2.0%)	6498 (3.0%)							
AIS in abdomen, median (IQR)	0 (0–3)	0 (0–0)	< 0.001	0.891	0 (0–3)		0 (0–2)		0.067
AIS of kidney, median (IQR)	0 (0–0)	0 (0–0)	< 0.001	0.157	0 (0–0)		0 (0–0)		0.033
Compartment syndrome in extremity, n (%)	85 (0.6%)	500 (0.2%)	< 0.001	0.057	80 (0.6%)		61 (0.5%)		0.020
*Treatment, n (%)*									
Laparotomy	1102 (7.8%)	5375 (2.5%)	< 0.001	0.242	1000 (7.9%)		1236 (9.7%)		0.066
Total Nephrectomy	43 (0.3%)	100 (0.0%)	0.004	0.062	35 (0.3%)		44 (0.3%)		0.013
Partial nephrectomy	2 (0.0%)	7 (0.0%)	0.102	0.012	2 (0.0%)		1 (0.0%)		0.007
Nephrorrhaphy	7 (0.0%)	27 (0.0%)	< 0.001	0.021	7 (0.1%)		11 (0.1%)		0.012
Thoracotomy	360 (2.5%)	1947 (0.9%)	< 0.001	0.126	325 (2.6%)		418 (3.3%)		0.043
RT	184 (1.3%)	1317 (0.6%)	< 0.001	0.071	161 (1.3%)		200 (1.6%)		0.026
REBOA	565 (4.0%)	697 (0.3%)	< 0.001	0.254	443 (3.5%)		355 (2.8%)		0.040
Vasopressors	1356 (9.6%)	5323 (2.5%)	< 0.001	0.302	1172 (9.2%)		1173 (9.2%)		0.000

*IQR* interquartile range, *CKD* chronic kidney disease, *BP* blood pressure, *GCS* Glasgow Coma Scale, *ISS* Injury Severity Score, *AIS* Abbreviated Injury Scale, *RT* resuscitative thoracotomy, *REBOA* resuscitative endovascular balloon occlusion of the aorta

**Table 2:** Details of angiography

Body region*, *n (%)*	
Head/neck	2411 (18.9%)
Chest	3126 (24.6%)
Abdomen/pelvis	10,624 (83.5%)
Others	1356 (10.7%)
Embolization	5541 (43.5%)

*Cases were double counted when angiography was conducted across more than two body regions

The propensity model was validated with 0.818 of c-statistic. Among the 14,180 patients in the angiography group, 12,724 patients were matched with controls in the non-angiography group. Patient characteristics after matching are summarized in Table 1. In the matched population, background characteristics of patients in the two groups became comparable (standardized differences were < 0.1 in all covariates after matching).

### Incidence of AKI and secondary outcomes

Unadjusted analysis identified that the incidence of AKI was significantly higher among patients who underwent emergency angiography compared to those who did not (160 [1.1%] vs. 537 [0.2%]; odds ratio [OR], 4.59; 95% confidence interval [CI], 3.85–5.48; *p* < 0.001; Table 3), and propensity score matching analysis revealed similar results (140 [1.1%] vs. 67 [0.5%]; OR, 2.10; 95% CI, 1.57–2.82; *p* < 0.001; Table 3). Hospital-free days and ICU-free days to day 30 were also shorter in patient in the angiography group than in those in the non-angiography group (2 [0–17] vs. 10 [0–22] days; difference in median, 0 [95% CI, 0–0] days; and 16 [0–25] vs. 20 [7–27] days; difference in median, 2 [95% CI, 1–2] days, respectively, Table 3).

**Table 3:** Angiography and clinical outcomes

	Angiography	No angiography	*p* value	OR (95% CI)		Difference in median (95% CI)	
AKI							
Unadjusted, *n (%)*	160 (1.1%)	537 (0.2%)	< 0.001	4.59	(3.85–5.48)		
PS matching, *n (%)*	140 (1.1%)	67 (0.5%)	< 0.001	2.10	(1.57–2.82)		
Hospital-free days to day 30, *days, median (IQR)*	2 (0–17)	10 (0–22)				0	(0–0)
ICU-free days to day 30, *days, median (IQR)*	16 (0–25)	20 (7–27)				2	(1–2)

*OR* odds ratio, *CI* confidence interval, *AKI* acute kidney injury, *PS* propensity score, *IQR* interquartile range, *ICU* intensive care unit

Inverse probability weighting analysis confirmed that the emergency angiography was associated with the higher incident of subsequent AKI (OR, 1.48; 95% CI, 1.32–1.67; Additional file 1: Table S1), and logistic regression with propensity score as a covariate identified the similar results (OR, 2.38; 95% CI, 1.86–3.04; Additional file 1: Table S1).

### Subgroup analysis

In the subgroup analyses (Table 4), the relationship between higher incidence of subsequent AKI and emergency angiography was observed in several subgroups: severe and mild/moderate injury, resuscitation with and without vasopressors, and early and late period of injury during the study period.

**Table 4:** In-hospital mortality in subgroup analyses

	Angiography	No angiography	OR	95% CI
Development of AKI				
with CKD	3.6% (0.0%–0.7%)	8.8% (0.0%–0.8%)	0.99	0.16–6.15
without CKD	1.1% (0.9%–1.3%)	0.5% (0.4%–0.6%)	2.13	1.58–2.86
Severe injury (ISS ≥ 25)	1.7% (1.4%–2.0%)	0.8% (0.6%–1.0%)	2.01	1.44–2.80
Mild/moderate injury (ISS < 25)	0.5% (0.3%–0.7%)	0.2% (0.1%–0.3%)	2.23	1.21–4.12
Treated with vasopressors	4.4% (3.2%–5.6%)	2.0% (1.2%–2.8%)	2.18	1.33–3.56
Treated without vasopressors	0.8% (0.6%–1.0%)	0.4% (0.3%–0.5%)	2.08	1.44–2.99
Older (age ≥ 65 years)	1.2% (0.9%–1.5%)	1.3% (0.8%–1.8%)	0.91	0.57–1.46
Younger (age < 65 years)	1.0% (0.8%–1.2%)	0.4% (0.3%–0.5%)	2.64	1.82–3.84
Early period (2004–2009)	1.7% (1.1%–2.3%)	0.4% (0.2%–0.6%)	4.25	2.46–7.32
Late period (2010–2018)	1.0% (0.8%–1.2%)	0.6% (0.4%–0.8%)	1.61	1.14–2.28

*OR* odds ratio, *CI* confidence interval, *AKI* acute kidney injury, *CKD* chronic kidney disease, *ISS* Injury Severity Scale

Conversely, patients who had CKD before injury had comparable incidence of AKI regardless of emergency angiography, whereas those without history of CKD developed AKI more frequently when they underwent emergency angiography (OR, 2.13; 95% CI, 1.58–2.86).

Furthermore, emergency angiography was associated with increased subsequent AKI among relatively younger patients who were aged < 65 years (OR, 2.64; 95% CI, 1.82–3.84), whereas the incidence of AKI was comparable between the angiography and non-angiography groups among those who were aged ≥ 65 years.

## Discussion

In this retrospective study, emergency angiography was associated with higher incidence of subsequent AKI. This relationship was validated even after several background risks for kidney injury and injury severity were adjusted. Thus, a future prospective study using predefined criteria for AKI is expected to be conducted for the validation of the current study.

Several pathophysiological mechanisms could be considered for the results in this study. First, the accumulated dose of contrast media is likely similar to the dosage used for coronary angiography (about 200 to 300 ml), and this amount of contrast is validated as a major risk factor for PC-AKI [22, 23]. During emergency angiography, radiologists usually use low-dose contrast (100 to 150 ml) [13] in an effort to avoid PC-AKI [22, 24]; however, most trauma patients would have undergone CT scan prior to angiography [25, 26] and received an additional 50 to 120 ml of contrast [13, 27]. Second, trauma victims who needed emergency angiography are bleeding, subsequently reducing intravascular volume. As decreased blood flow in the renal arteries precipitates kidney injury [10], the addition of contrast presents an increased risk of nephrotoxicity. Third, systemic inflammation following severe injury affects the tolerance for kidney insult by contrast media. Given that increased acute inflammation is associated with increased risk of PC-AKI [28], background risks of AKI would be high among patients in this study.

In the subgroup analyses, patients with a history of CKD did not have higher incidence of AKI after exposure to the contrast media, which is different from previous studies [29, 30]. One of the possibilities would be potential differences in procedures of angiography between patients with and without CKD. In Japan, standard practice by radiologists in the setting of CKD is to utilize alternative contrast agents [31, 32], and therefore in these cases radiologists would have used less-nephrotoxic agent such as carbon dioxide, instead of contrast media, when baseline kidney function was severely compromised. It should be also emphasized that the 95% CI of OR for developing AKI was wide (0.16–6.15) and including more patients with CKD would possibly reach a different result. Moreover, the length of hospital stay (LOS) was comparable between the angiography and non-angiography groups, although past studies suggested that PC-AKI was associated with prolonged hospital stay [33]. Considering that the incidence of subsequent AKI was rare in this study, the small number of patients with AKI might not have significantly affected LOS in all the patients. Notably, the length of ventilator usage was longer by 1–2 days in patients treated with the angiography.

Some potential preventions for PC-AKI can be considered if emergency angiography is validated as an independent risk for PC-AKI by a future study. Given that preprocedural hydration with saline or bicarbonate has been shown to prevent PC-AKI [34–36], restoration of intravascular volume should be achieved before emergency angiography. As anti-inflammatory medications, such as statins, have shown promising results for the prevention of PC-AKI in clinical studies on coronary angiography [37], this potential treatment should be investigated as an adjunct in trauma patients who require an angiography. Urine alkalization is scheduled to be investigated in an upcoming randomized controlled trial and may become an option for PC-AKI prevention in the near future [38]. To define clinical benefits of such managements, the results in the current study should be further validated in prospective studies using uniform criteria for the diagnosis of AKI.

The results in this study must be interpreted within the context of the study design. We investigated data using the JTDB, which does not record details of emergency angiography, including the indication for the procedure. Thus, our results could have been different if the decision for emergency angiography had been dependent on unrecorded strong prognostic factors for AKI. Another limitation is that serum creatinine, urine output, and hemodialysis requirement were not available in the database. Although AKI was diagnosed and recorded by treating physicians according to published clinical criteria, the specific criteria used for AKI definition and/or the degree of AKI severity could not be evaluated in this study: As this is a significant limitation, a future prospective study must be conducted to validate the current results. Moreover, we defined the primary outcome as AKI that was subsequently developed after emergency angiography, regardless of the timing of the diagnosis. As CIN, CI-AKI, and PC-AKI are usually defined as newly developed or worsened kidney dysfunction within 2–3 days after the exposure to contrast media [9], our results would be different if such standard definitions are used. However, as no changes in serum creatinine and/or urine output within a few days would not deny the possibility of gradually deteriorating kidney dysfunction [39], examining the incidence of AKI for longer period would be more clinically relevant. Finally, we investigated only emergency angiographies that were urgently performed during the initial resuscitation. Therefore, the results in this study should not be applied to scheduled angiography, such as one performed for a pseudoaneurysm found later during the hospital course.

## Conclusions

Emergency angiography was associated with increased incidence of subsequent AKI among trauma patients. This result should be validated in a future study using predefined criteria for the diagnosis of AKI. Prevention measures for PC-AKI, such as preprocedural hydration, should be considered in the setting of emergency angiography.

## Supplementary Information

Additional file 1: Table S1. Development of AKI in sensitivity analyses.

## Data Availability

The data of this study are available from the Japanese Association for Trauma Surgery and the Japanese Association for Acute Medicine; however, restrictions apply to the availability of these data, which were used under the license for the current study and so are not publicly available. However, data are available from the authors upon reasonable request and with permission of the Japanese Association for Trauma Surgery and the Japanese Association for Acute Medicine.

## Abbreviations

AKI: Acute kidney injury
ISS: Injury Severity Scale
CIN: Contrast-induced nephropathy
CI-AKI: Contrast-induced AKI
PC-AKI: Post-contrast AKI
JTDB: Japan Trauma Data Bank
AIS: Abbreviated Injury Scale
ISS: Injury Severity Score
RT: Resuscitative thoracotomy
REBOA: Resuscitative endovascular balloon occlusion of the aorta
ICU: Intensive care unit
CKD: Chronic kidney disease
CI: Confidence interval
GCS: Glasgow Coma Scale
sBP: Systolic blood pressures
OR: Odds ratio
