# Supplementary material for: Emergency angiography for trauma patients and potential association with acute kidney injury
Source: World J Emerg Surg. 2021 Nov 4;16:56. doi: 10.1186/s13017-021-00400-0 (PMC8567733; doi:10.1186/s13017-021-00400-0)
Supplement: Supplementary file 1 — Development of AKI in sensitivity analyses. [file 13017_2021_400_MOESM1_ESM.docx]

| Table S1. Development of AKI in sensitivity analyses | | | |  |
| --- | --- | --- | --- | --- |
|  |  | OR | 95% CI |  |
| Development of AKI | |  |  |  |
|  | IPW | 1.48 | 1.32–1.67 |  |
|  | Regression with propensity score | 2.38 | 1.86–3.04 |  |
| AKI = acute kidney injury, OR = odds ratio, CI = confidence interval, and IPW = inverse probability weighting. | | | |  |
|  |  |  |  |  |
|  |  |  |  |  |
